# Supplementary material for: Skeletonization of neuronal processes using Discrete Morse techniques from computational topology
Source: Res Sq. 2025 Jun 20:rs.3.rs-6642891. Preprint. [Version 1] doi: 10.21203/rs.3.rs-6642891/v1 (PMC12204360; doi:10.21203/rs.3.rs-6642891/v1)
Supplement: Supplement 1 [file NIHPPrs6642891v1-supplement-1.pdf]

Supplementary Files

This is a list of supplementary files associated with this preprint. Click to download.

- S2SupplementalPSF.pdf
- S1SupplementalDMTutorial.pdf
- SkeletonizationExtendedDataFigure.pdf
